# Toll-Like Receptor 2 Release by Macrophages: An Anti-inflammatory Program Induced by Glucocorticoids and Lipopolysaccharide

**DOI:** 10.3389/fimmu.2019.01634

**Published:** 2019-07-23

**Authors:** Jessica Hoppstädter, Anna Dembek, Rebecca Linnenberger, Charlotte Dahlem, Ahmad Barghash, Claudia Fecher-Trost, Gregor Fuhrmann, Marcus Koch, Annette Kraegeloh, Hanno Huwer, Alexandra K. Kiemer

**Affiliations:** ^1^Department of Pharmacy, Pharmaceutical Biology, Saarland University, Saarbrücken, Germany; ^2^Department of Computer Science, German Jordanian University, Amman, Jordan; ^3^Department of Experimental and Clinical Pharmacology and Toxicology, Saarland University, Homburg, Germany; ^4^Helmholtz Institute for Pharmaceutical Research Saarland, Saarbrücken, Germany; ^5^INM—Leibniz Institute for New Materials, Saarbrücken, Germany; ^6^Department of Cardiothoracic Surgery, Völklingen Heart Centre, Völklingen, Germany

**Keywords:** innate immunity, corticosteroid, pulmonary macrophage, exosome, microvesicle

## Abstract

Glucocorticoids (GCs) are widely prescribed therapeutics for the treatment of inflammatory diseases, and endogenous GCs play a key role in immune regulation. Toll-like receptors (TLRs) enable innate immune cells, such as macrophages, to recognize a wide variety of microbial ligands, thereby promoting inflammation. The interaction of GCs with macrophages in the immunosuppressive resolution phase upon prolonged TLR activation is widely unknown. Treatment of human alveolar macrophages (AMs) with the synthetic GC dexamethasone (Dex) did not alter the expression of TLRs −1, −4, and −6. In contrast, TLR2 was upregulated in a GC receptor-dependent manner, as shown by Western blot and qPCR. Furthermore, long-term lipopolysaccharide (LPS) exposure mimicking immunosuppression in the resolution phase of inflammation synergistically increased Dex-mediated TLR2 upregulation. Analyses of publicly available datasets suggested that TLR2 is induced during the resolution phase of inflammatory diseases, i.e., under conditions associated with high endogenous GC production. TLR2 induction did not enhance TLR2 signaling, as indicated by reduced cytokine production after treatment with TLR2 ligands in Dex- and/or LPS-primed AMs. Thus, we hypothesized that the upregulated membrane-bound TLR2 might serve as a precursor for soluble TLR2 (sTLR2), known to antagonize TLR2-dependent cell actions. Supernatants of LPS/Dex-primed macrophages contained sTLR2, as demonstrated by Western blot analysis. Activation of metalloproteinases resulted in enhanced sTLR2 shedding. Additionally, we detected full-length TLR2 and assumed that this might be due to the production of TLR2-containing extracellular vesicles (EVs). EVs from macrophage supernatants were isolated by sequential centrifugation. Both untreated and LPS/Dex-treated cells produced vesicles of various sizes and shapes, as shown by cryo-transmission electron microscopy. These vesicles were identified as the source of full-length TLR2 in macrophage supernatants by Western blot and mass spectrometry. Flow cytometric analysis indicated that TLR2-containing EVs were able to bind the TLR2 ligand Pam_3_CSK_4_. In addition, the presence of EVs reduced inflammatory responses in Pam_3_CSK_4_-treated endothelial cells and HEK Dual reporter cells, demonstrating that TLR2-EVs can act as decoy receptors. In summary, our data show that sTLR2 and full-length TLR2 are released by macrophages under anti-inflammatory conditions, which may contribute to GC-induced immunosuppression.

## Introduction

Glucocorticoids (GCs) represent the most effective anti-inflammatory drugs in the therapy of inflammatory lung diseases. Genes that are upregulated by GC treatment, such as dual-specificity phosphatase 1 (DUSP1), GC-induced leucine zipper (GILZ), and interleukin (IL)-10, are highly immunosuppressive and contribute to the overall effect of GC treatment (1–3).

Alveolar macrophages (AMs) are the tissue-resident macrophages in the lung alveolar space. They represent the first line of defense against pathogens in the lower airspace and recognize microbial ligands *via* pattern recognition receptors (4, 5). Toll-like receptors (TLRs) are the major pattern recognition receptors of the innate immune system that sense a wide range of “danger” signals or pathogen-associated molecular patterns (PAMPs) (6–8).

To date, 10 TLRs have been identified in humans. Surface-expressed TLRs (i.e., TLR1, −2, −4, −5, −6, and −10) recognize bacterial, fungal, and parasitic PAMPs, whereas endosomal TLRs (i.e., TLR3, −7/−8, and −9) sense nucleic acids of viral or bacterial origin. After recognition and binding of a specific PAMP, TLRs induce an intracellular signaling cascade that culminates in the activation of the activator protein (AP)-1, nuclear factor (NF)-κB, and interferon regulatory factors (IRFs). These signaling cascades result in the secretion of pro-inflammatory factors that ultimately protect the host from microbial infection (6, 9).

Although GCs usually dampen TLR signaling, GC-mediated induction of TLR2 has for example been shown in dendritic cells (10), THP-1 macrophages (11), and AMs (12). TLR2 recognizes a wide variety of pathogens, including bacteria, viruses, fungi, mycobacteria, and parasites. Unlike other TLRs, the formation of TLR2 heterodimers with other TLR family members (i.e., TLR1, TLR6, or TLR10) or non-TLR cellular molecules (e.g., CXCR4 or scavenger receptors) is a prerequisite for the initiation of cell activation (13).

Since TLR2 activity plays a prominent role in the pathogenesis of numerous acute and chronic inflammatory diseases, its activation has to be tightly regulated. In general, negative regulation of TLR signaling can be mediated by soluble factors, including soluble TLRs (sTLR) that act as decoy receptors and bind to PAMPs in the extracellular space, preceding their engagement with specific PRRs and reducing TLR signaling efficiency (14–16). sTLR2 is produced *via* proteolytic cleavage of the TLR2 trans-membrane protein, also referred to as ectodomain shedding, by disintegrin metalloproteinases (ADAMs) (17). Elevated sTLR2 plasma levels were observed in experimental models of human endotoxemia and sepsis patients and have therefore been suggested as a biomarker for infections (18, 19).

Sepsis represents a life-threatening systemic inflammation caused by bacterial infections. If sepsis patients survive the acute inflammatory response, compensatory mechanisms result in profound immunosuppression, often leading to lethal secondary infections. Several critical factors have been identified that contribute to the transition of the pro-inflammatory phase into the immunosuppressive phase, including endogenous GCs (1, 20, 21). In addition, prolonged exposure to bacterial components, such as lipopolysaccharide (LPS), skews monocytes and macrophages toward a hypo-responsive state termed LPS tolerance. LPS-tolerant cells are characterized by a decreased ability to produce pro-inflammatory mediators whereas their expression of mediators involved in immunosuppression and wound healing is elevated (20).

In the present study, we examined TLR2 expression in primary human AMs after GC administration and in chronic inflammation, as mimicked by prolonged LPS treatment.

## Materials and Methods

### Materials

RPMI1640 (#R0883), DMEM (#D6546), trypsin/EDTA (#T3924), fetal calf serum (FCS, #F7524), penicillin / streptomycin (#P433), kanamycin (#K0254), and glutamine (#G7513) were from Sigma-Aldrich. Endothelial cell growth media (#C-22010) including supplement mix (#C-39215) were from PromoCell. The anti-TLR2 antibody used for Western blot analysis was obtained from Abcam (EPNCIR133, #ab108998). The Phospho-p38 MAPK (Thr180/Tyr182, 3D7, #9215) and total p38 MAPK (#9212, polyclonal) antibodies were from Cell Signaling. The anti-tubulin antibody (#T9026) was obtained from Sigma-Aldrich. Anti-rabbit IRDye 680- and anti-mouse IRDye 800-conjugated secondary antibodies were from LI-COR Biosciences (#926-68071, #926-32210). The anti-rabbit IRDye 800-conjugated secondary antibody was from Rockland (#612-132-120). APC-labeled anti-TLR2 and the respective isotype control were from ThermoFisher Scientific (TL2.1, # 17-9922-41; IgG2a kappa Isotype Control #17-4724-81). FITC anti-CD9 (HI9a, #BLD-312103), FITC anti-CD63 (H5C6, #BLD-353005), and the respective isotype control (MOPC-21, #BLD-400109) were purchased from Biozol. The Zombie Yellow^TM^ Fixable Viability Kit (#423103) was from BioLegend. Ultrapure LPS from *Escherichia coli* K12 (#tlrl-peklps), Pam_3_CSK_4_ (#tlrl-pms), rhodamine-labeled Pam_3_CSK_4_ (#tlrl-rpms), Pam_2_CSK_4_ (#tlrl-pm2s), heat-killed *Staphylococcus aureus* (#tlrl-hksa), lipoteichoic acid (LTA, # tlrl-pslta), normocin (#ant-nr-1), and zeocin (#ant-zn-1) were obtained from Invivogen. Phorbol 12-myristate 13-acetate (PMA, # 524400) was from Cayman Chemical. Dexamethasone (#D8893) was obtained from Sigma-Aldrich. Dexamethasone stock solutions were either prepared in DMSO or ethanol (EtOH), and the appropriate vehicle control is indicated in the figure legends. Alternatively, water-soluble dexamethasone 21-phosphate disodium salt (Sigma-Adrich, #D1159) was dissolved in medium, and untreated cells served as a control (**Figures 6C,D**, **7**, and **8**). Primers and dual-labeled probes were from Eurofins MWG Operon. Taq polymerase (5 U/μL, #E00007), Taq buffer (#B0005), and the dNTP mix (#D0056) were from Genscript. Other chemicals were obtained from either Sigma-Aldrich or Carl Roth unless stated otherwise.

### Cell Culture

#### Cell Lines

THP-1 (#TIB202) and L929 cells (#CRL-6364) were obtained from ATCC and grown in RPMI 1640 supplemented with 10% FCS, 100 U/mL penicillin G, 100 μg/mL streptomycin, and 2 mM glutamine. THP-1 were differentiated into macrophage-like cells by treatment with PMA (100 nM) for 48 h. HEK-Dual™ hTLR2 reporter cells (Invivogen, #hkd-htlr2ni) were grown in DMEM supplemented with 10% FCS, 2 mM glutamine, 50 U/mL penicillin G, 50 μg/mL streptomycin, 100 μg/mL normocin, and 100 μg/mL zeocin.

#### Human Alveolar Macrophages (AMs)

Human lung tissue was obtained from patients undergoing lung resection. The use of human material was reviewed and approved by the local ethics committee (State Medical Board of Registration, Saarland, Germany; permission no. 213/06). The informed consent of all participating subjects was obtained. AM isolation was performed according to a previously described method (4, 22, 23) with minor modifications. After visible bronchi were removed, the lung tissue was chopped and washed with PBS (137 mM NaCl, 2.7 mM KCl, 10.1 mM Na_2_HPO_4_, 1.8 mM KH_2_PO_4_, pH 7.4). The washing buffer was collected and centrifuged (15 min, 350 x g). Remaining erythrocytes were lysed by briefly resuspending the pellet in autoclaved water, followed by immediate washing with PBS and centrifugation. Cells were resuspended in AM medium (RPMI 1640 containing 5% FCS, 100 U/mL penicillin G, 100 μg/mL streptomycin, and 2 mM glutamine). Unless stated otherwise, AMs were seeded at a density of 0.5–1 × 10^6^ cells/well into a 12- or 6-well plate and incubated at 37°C for 2 h, washed with PBS, and cultured overnight before further use. AM preparations were 95% pure as judged by flow cytometric analysis of intracellular CD68 (4, 24).

#### Human Umbilical Vein Endothelial Cells (HUVECs)

HUVECs were isolated from umbilical cords provided by the Klinikum Saarbrücken (Saarbrücken, Germany; ethics committee permission no. 131/08). The informed consent of all donors was obtained. HUVEC isolation and culture was performed as described previously (25, 26). In brief, HUVECs were isolated by digestion of umbilical veins with 100 mg/L collagenase A (Roche, Mannheim, Germany). Cells were grown in endothelial growth medium with supplement mix, 100 U/mL penicillin G, 100 μg/mL streptomycin, 50 mg/mL kanamycin, and 10% FCS. For all experimental procedures, HUVECs were used in passage three. Cells were detached with trypsin/EDTA, seeded at a density of 1 × 10^5^ cells per well in a 24-well plate and incubated overnight before further treatment. HUVECs were >95% pure, as assessed by flow cytometry using an antiserum against the von Willebrand factor (27).

### TNF-α Bioassay

TNF-α concentrations in cell culture supernatants were quantified by bioassay as previously described (28). L929 cells were seeded into a 96-well plate (3 × 10^4^ cells per well) and incubated overnight at 37°C, 5% CO_2_. The medium was discarded, and 100 μL of actinomycin D solution (1 μg/mL in growth medium) was added. After incubation for 1 h at 37°C, AM supernatants (100 μL per well) were added. Dilution series of recombinant human TNF-α (100–2,500 pg/mL) were run alongside the samples to generate a standard curve. The plate was incubated for 24 h at 37°C, followed by incubation with MTT solution (0.5 mg/mL in medium) for 2 h. The supernatant was discarded, and cells were lysed in 100 μL DMSO. Absorbance measurements were carried out at 550 nm with 630 nm as the reference wavelength using a microplate reader (Tecan Sunrise).

### RNA Isolation, Reverse Transcription, and Quantitative RT-PCR

Total RNA was isolated using the RNeasy Plus Mini Kit (Qiagen, #74134) or the High Pure RNA Isolation Kit (Roche, # 11828665001), and RNA was reverse transcribed using the High-Capacity cDNA Reverse Transcription Kit (Applied Biosystems, #4368813) according to the manufacturer's instructions. The cDNA was diluted with TE buffer (Applichem, #A0386) before use. The CFX96 Touch™ Real-Time PCR Detection System (Bio-Rad) was used for real-time RT-PCR. For *ACTB, CXCL10, IL10, TLR1, TLR2, TLR4, TLR6*, and *TNF*, one 25 μL reaction mix contained 2.5 U Taq polymerase, 500 nM sense and antisense primers, 60-100 nM probe, 200 μM dNTPs, 3-4 mM MgCl_2_, 2.5 μL 10x Taq buffer, 3 μL Template, and molecular biology grade water (Applichem, #A7398). The reaction conditions were 95°C for 8 min followed by 40 cycles of 15 s at 95°C, 15 s at a reaction dependent temperature varying from 57 to 60°C, and 15 s at 72°C. For *ADAM10, ADAM17, CCL2, DUSP1, FPR2, ICAM, MMP9, SELE*, and *VCAM* detection, the 5x HOT FIREPol® EvaGreen® qPCR Mix Plus (Solis Biodyne, #08-25) was used according to the manufacturer's recommendations. Primer and probe sequences, as well as specific reaction conditions, are given in Supplementary Table 1. Standard curves were generated by using a dilution series of the PCR product cloned into pGEMTeasy (Promega, #A1360) (23, 28, 29). All samples and standards were analyzed in triplicate. All samples were normalized to the housekeeping gene *ACTB*.

### Extracellular Vesicles (EVs)

#### Isolation

AMs or differentiated THP-1 cells were incubated for 3 d in FCS-free medium in the presence or absence of LPS (100 ng/mL) and/or dexamethasone (1 μM). EVs were purified from cell culture supernatants by sequential centrifugation as previously described (30). For THP-1-derived vesicles, 5 × 10^7^ cells were used per preparation. After differentiation for 48 h, cells were washed with PBS, and FCS-free medium was added. Serum deprivation did not result in increased cell death, as indicated by caspase 3 assay and Zombie Yellow staining (Supplementary Figure 1).

Cell culture supernatants were collected and centrifuged at 300 × g for 10 min to remove remaining cells, followed by removal of dead cells and large cell debris by centrifugation at 2,000 × g for 10 min and 10,000 × g for 30 min. Supernatants were transferred into stable polycarbonate tubes (# 4416, Laborgeräte Beranek), and EVs were collected by ultracentrifugation at 100,000 x *g* for 90–120 min in an L70 ultracentrifuge with a 70Ti rotor (Beckman Coulter). EVs were washed with 25 mL sterile-filtered PBS and pelleted again by ultracentrifugation (100,000 × g, 90–120 min). The EV pellet was then resuspended in sterile-filtered PBS (AMs: 200–350 μL; THP-1: 200 μL) and stored at −80°C in protein LoBind microcentrifuge tubes (# Z666505, Eppendorf).

#### Nanoparticle Tracking Analysis (NTA)

For nanoparticle tracking analysis (NTA), EV suspensions were diluted 1:200 in sterile-filtered PBS. 300–500 μL of the dilution were injected into the sample chamber of a NanoSight LM10 (NanoSight Ltd). A video of 60 s was recorded and analyzed by the NTA software Nanosight NTA 2.3 to calculate vesicle size and concentration.

#### Protein Concentration

Total protein concentrations were determined with the Pierce BCA protein assay kit (ThermoFisher Scientific, #23225) using a GloMax® Discover Multimode Microplate Reader (Promega) according to the manufacturer's instructions.

#### Cryo-Transmission Electron Microscopy (TEM)

A 3 μL droplet of the aqueous EV dilution was placed onto a holey carbon covered TEM grid (Plano, type S147-4), plotted onto a thin liquid film for 2 s and plunged into a bath of liquid ethane at −165°C using a Gatan CP3 cryoplunger (Pleasanton). The frozen sample was transferred under liquid nitrogen to a Gatan cryo-TEM sample holder (model 914) and investigated at −173°C by low-dose bright-field imaging TEM (JEOL JEM-2100 LaB6). A Gatan Orius SC1000 CCD camera was used for image acquisition.

#### Proteomics

Thirty micrograms of EV protein were precipitated by trichloroacetic acid (TCA) precipitation with an end concentration of 20% TCA. Samples were washed thrice with acetone. After a final centrifugation of 15 min in a SeedVac Plus concentrator (Savant, Thermo Fisher, Waltham, USA), samples were resuspended in 2x Lämmli buffer (4% SDS, 20% glycerol, 120 mM Tris-HCl (pH 6.8), 0.02% bromophenol blue in Millipore water) and denatured at 95°C for 5 min. Proteins were separated on NuPAGE® 10% gels and prepared for mass spectrometry as described previously (31). Three protein bands per sample were cut out of the gel and incubated with porcine trypsin (Promega, #V5111) for in-gel digestion at 37°C overnight. Resulting peptides were extracted twice by shaking the gel pieces in aqueous extraction buffer (2.5% formic acid, 50% acetonitrile). Extracted peptides were concentrated via vacuum centrifugation and resuspended in 0.1% formic acid. Six microliters of each tryptic peptide extract were analyzed by online nanoflow LC-HR-MS/MS (Ultimate 3000 RSLC nano system equipped with an Ultimate3000 RS autosampler coupled to an LTQ Orbitrap Velos Pro, ThermoFisher Scientific) as described previously (31). Peptides were analyzed at a flow rate of 200 μL/min with buffer A (water and 0.1% formic acid) and B (90% acetonitrile and 0.1% formic acid) using the gradient given in Supplementary Figure 2. Fragmented peptides were identified using software Proteome Discoverer 1.4 (ThermoFisher Scientific) and database SwissProt 2015_01 (species human). For further data evaluation, software Scaffold4 (version 4.8.3) was used. In order to allow expression of x-fold values if a protein was absent in one of the treatments, log2 fold changes were calculated as log2[(mean of unique spectrum counts in EV_LPS+Dex_) + 0.1) / (mean of unique spectrum counts in EV_Co_ + 0.1)]. The mass spectrometry proteomics data have been deposited to the ProteomeXchange Consortium via the PRIDE partner repository (32) with the dataset identifier PXD013977 and 10.6019/PXD013977.

#### Flow Cytometry

For analysis of EV surface proteins by flow cytometry, vesicles were coupled to the surface of 4 μm aldehyde/sulfate latex beads (Invitrogen, #A37304). In detail, an amount of EVs resembling 10 μg protein or the same amount of the negative control BSA were allowed to bind to 10 μL latex beads for 15 min at room temperature in a final volume of 100 μL in PBS. After adding 400 μL PBS, samples were incubated for 1 h at room temperature with gentle shaking. The reaction was stopped by adding 500 μL 200 mM glycine, followed by incubation for 30 min at room temperature. EV- or BSA-coupled beads were washed three times with 1% BSA in PBS, with centrifugation steps at 2,000 × g for 3 min in between. Samples were stained with fluorescently labeled antibodies directed against TLR2 or the EV markers CD9 and CD63 or the respective isotype controls on ice in the dark. Staining with rhodamine-labeled Pam_3_CSK_4_ was performed accordingly. Details are given in Supplementary Table 2. After 30 min, samples were washed twice with 1% BSA in PBS and analyzed on a BD LRS Fortessa (BD Biosciences) using BD FACSDiva 8.0. For graphical illustrations, BD FACSuite (version 1.0) software was used.

### Western Blotting

For whole cell analysis, cells were lysed in lysis buffer (50 mM Tris-HCl, 1% (m/v) SDS, 10% (v/v) glycerol, 5% (v/v) 2-mercaptoethanol, 0.004% (m/v) bromphenol blue) supplemented with a protease inhibitor mix (cOmplete; Roche Diagnostics, #04693124001). Samples were sonicated, centrifuged at 10,000 × g for 10 min at 4°C, and stored at −80°C until further use (23, 28, 29, 33). Cell culture supernatants from AMs cultured in a 12 well plate (5 × 10^5^ cells per well in 300 μL medium) were concentrated 10x by centrifugation at 15,000 × g for 8 min in Vivaspin®500 tubes with 10 kDa cut off (Sartorius #VS0102). Concentrated supernatants (21 μL per lane), as well as isolated EVs (5 × 10^9^ vesicles per lane), were supplemented with a 4x loading buffer (Carl Roth, Roti®-load 1, #K929.1). Before gel electrophoresis, all samples were denatured at 95°C for 5 min and subsequently kept on ice before gel loading.

SDS-polyacrylamide gel electrophoresis (PAGE) was carried out using polyacrylamide gels (4% stacking gel, 12% resolving gel) and the Mini-PROTEAN®system (Bio-Rad). A prestained protein ladder was used to estimate the molecular mass (#26616, ThermoFisher Scientific). Samples were transferred onto an Immobilon FL-PVDF membrane (# IPFL00010, Millipore-Merck, Darmstadt, Germany) using a Mini Trans-Blot®Cell (Bio-Rad). The membrane was blocked for 1–4 h at room temperature in blocking buffer for near-infrared fluorescent Western blotting (#MB-070, Rockland) to saturate unspecific binding sites. Subsequently, the membrane was incubated with primary antibody dilutions (1:500–1:2,000 in Rockland blocking buffer) at 4°C, either overnight or for 48 h. After thorough washing with PBST (PBS + 0.1% Tween-20), the membrane was stained with IRDye680- or IRDye800-conjugated secondary antibodies (1:5,000–1:10,000) diluted in blocking buffer for 1.5-2 h at room temperature, washed again, and signals were detected and quantified using an Odyssey imager and software (LI-COR Biosciences). For densitometric analysis, signal intensities were normalized to the loading control tubulin except for pp38 which was normalized to values for total p38.

### Viability Assays

#### Caspase 3-Like Assay

Cells were washed twice with ice-cold PBS. Seventy microliters ice-cold lysis buffer (25 mM HEPES, 5 mM MgCl_2_, 1 mM EGTA, 0.1% [v/v] Triton X-100) were added, and the samples were stored at −80°C. After thawing on ice, the lysates were centrifuged (14,000 × g, 10 min, 4°C) and 10 μL of the supernatant were transferred to a black 96 well plate (TPP). 90 μL substrate solution (55 μM of fluorogenic substrate Ac-DEVD-AFC (Enzo, #ALX-260-032-M005) 50 mM HEPES, 0.1% [w/v] CHAPS, 1% [w/v] sucrose, 10 mM DTT, pH 7.5) were added, and the production of free 7-amino-4-trifluoro-methyl coumarin (AFC) at 37°C was determined by fluorescence measurement (excitation: 405 nm; emission: 495-505 nm) using a GloMax® Discover Multimode Microplate Reader (Promega).

#### Zombie Yellow Staining

Cells were stained with the Zombie Yellow^TM^ Fixable Viability Kit as recommended by the supplier. Samples were analyzed on a BD LRS Fortessa (BD Biosciences) using BD FACSDiva 8.0 software.

### HEK-Dual hTLR2 Reporter Assay

HEK-Dual™ hTLR2 reporter cells express TLR2, an NF-κB/AP1-inducible secreted embryonic alkaline phosphatase (SEAP) reporter gene, and a secretable luciferase reporter gene (Lucia luciferase) placed under the control of the endogenous IL-8 promoter.

Cells were seeded into 96-well plates (5 × 10^5^ cells/well) and immediately treated as indicated to monitor TLR2-dependent activation. After 24 h, supernatants were collected, and the activity of Lucia luciferase was determined using the QuantiLuc reagent (Invivogen, #rep-qlc1) according to the supplier's instructions. SEAP activity could not be used as a readout parameter in our setting because EVs interfered with the assay (data not shown).

### Analysis of Publicly Available Datasets

Datasets were obtained from Gene Expression Omnibus (GEO) and normalized using log2-RMA. Dataset GSE4607 included transcriptional profiles human whole blood samples of 15 healthy controls, 27 patients with non-infectious SIRS, and 12 samples from patients with resolved non-infectious SIRS. Patients were classified as SIRS or SIRS resolved (no longer meeting criteria for SIRS) on d3 after ICU admittance. Dataset GSE8121 included transcriptional profiles of human whole blood samples of 15 healthy controls and 30 patients with sepsis. Samples were obtained at d1 and d3 after ICU admission. Statistical significances were determined by the Kolmogorov–Smirnov test. Detailed information about the patient cohort is given in the GEO database and the corresponding original publications (34, 35).

### Statistics

All experiments were performed at least three times, and at least two replicates were analyzed for all experiments unless stated otherwise. Data distribution was determined by the Shapiro-Wilk test. For normally distributed data, means of two groups were compared with non-paired two-tailed Student's *t*-test or one sample *t*-test where applicable. For data that were not normally distributed, means of two groups were compared using the Mann-Whitney test. Means of more than two groups were compared by one-way ANOVA with Bonferroni's *post hoc* test (normal distribution) or Kruskal–Wallis ANOVA followed by Mann-Whitney test (no normal distribution). Statistical significance was set at *p* < 0.05, *p* < 0.01, or *p* < 0.001. Data analysis was performed using Origin software (OriginPro 2015G; OriginLab).

## Results

### Upregulation of TLR2 by GCs

**Figure 1:**
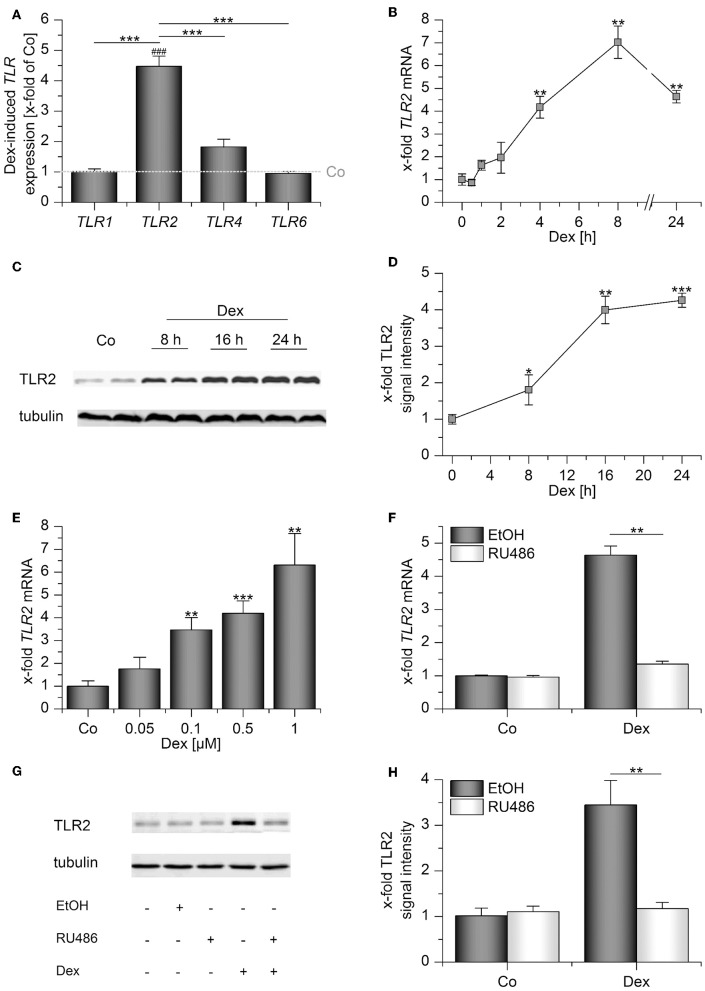
Dexamethasone induces TLR2 in AMs. **(A–E)** AMs were incubated with solvent control (0.1% DMSO, Co) or dexamethasone (Dex, 1 μM) for up to 24 h **(A–D)** or at the indicated concentrations for 4 h **(E)**. **(E–G)** AMs were preincubated with the GR inhibitor RU486 (10 μM) or solvent control (0.1% EtOH) and treated with Dex (1 μM) for 24 h. Data from at least three independent experiments performed in duplicate with cells from different donors are presented as means ± SEM. TLR expression was measured by or qPCR **(A,B,E,F)** or Western blot **(C,D,G,H)**. **(A)** TLR expression upon Dex treatment was normalized to the TLR expression values for the respective vehicle-treated control (indicated by the dotted line). **(B–H)** TLR2 expression in solvent-treated cells were set as 1. **(C,G)** Representative blots. **(D,H)** Densitometric analysis. TLR2 signal intensities were quantified and normalized to the loading control tubulin. ^*^p < 0.05, ^**^p < 0.01, ^***^p < 0.001, ^*###*^*p* < 0.001 vs. vehicle-treated cells. *p*-values were generated by ANOVA with Bonferroni's *post-hoc* test or Mann–Whitney *U*-test.

Human AMs express the surface TLRs −1, −2, −4, and −6 (4). Thus, we initially quantified the expression of these receptors after treatment with the GC dexamethasone (Dex) for 24 h by qPCR. Whereas Dex administration had no significant effect on *TLR1*, −*4*, and −*6* mRNA levels, *TLR2* was highly induced (Figure 1A). Further analysis showed that *TLR2* upregulation was already detectable 4 h after treatment (Figure 1B), and TLR2 protein production was maximal after 16 h, as shown by Western blot analysis (Figures 1C,D). Dex induced *TLR2* starting at a concentration of 100 nM (Figure 1E). Next, we evaluated whether Dex binding to the GR is necessary for TLR2 induction. To this end, we pretreated AMs with RU486, a specific GR antagonist, before Dex was added. RU486 completely abrogated Dex-mediated *TLR2* mRNA and TLR2 protein upregulation, indicating a GR-dependent mechanism (Figures 1F–H).

### TLR2 Induction in SIRS and Sepsis

**Figure 2:**
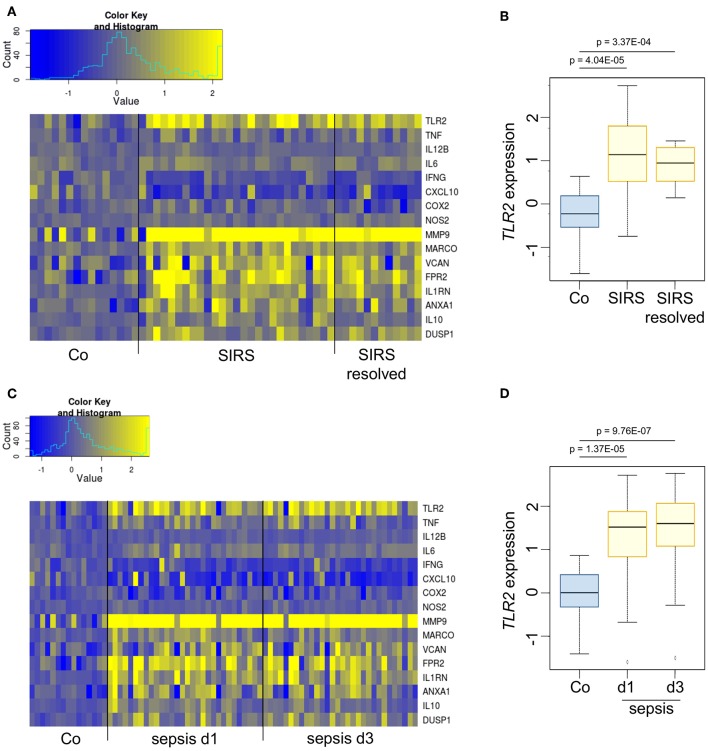
TLR2 is overexpressed during the immunosuppressive phases of SIRS and sepsis. **(A,B)** Dataset GSE4607 was obtained from Gene Expression Omnibus (GEO) and normalized using log2-RMA. The dataset included transcriptional profiles of human whole blood samples of 15 healthy controls, 27 patients with non-infectious SIRS and 12 samples from patients with resolved non-infectious SIRS. Patients were classified as SIRS, or SIRS resolved (no longer meeting criteria for SIRS) on d3 after ICU admittance. **(C,D)** Dataset GSE8121 was retrieved from GEO and normalized using log2-RMA. The dataset included transcriptional profiles of human whole blood samples of 15 healthy controls and 30 patients with sepsis. Samples were obtained at d1 and d3 after admittance to the ICU. The statistical significance was determined by the Kolmogorov–Smirnov test.

*In vivo*, endogenous GCs contribute to immunosuppression occurring at later stages of inflammatory processes (1, 20, 21). Analyses of publicly available datasets showed that *TLR2* mRNA was induced in whole blood samples from pediatric patients suffering from SIRS (Figures 2A,B) or sepsis (Figures 2C,D). In both groups, *TLR2* induction was paralleled by the upregulation of genes involved in the resolution of inflammation or wound healing (*MMP9, MARCO, VCAN, FPR2, IL1RN, ANXA1, IL10, DUSP1*), whereas the gene expression of pro-inflammatory factors (*TNF, IL12B, IL6, IFNG, CXCL10, COX2, NOS2*) was not elevated compared with healthy controls, suggesting the onset of anti-inflammatory feedback mechanisms.

### TLR2 Induction by LPS in the Absence or Presence of GCs

**Figure 3:**
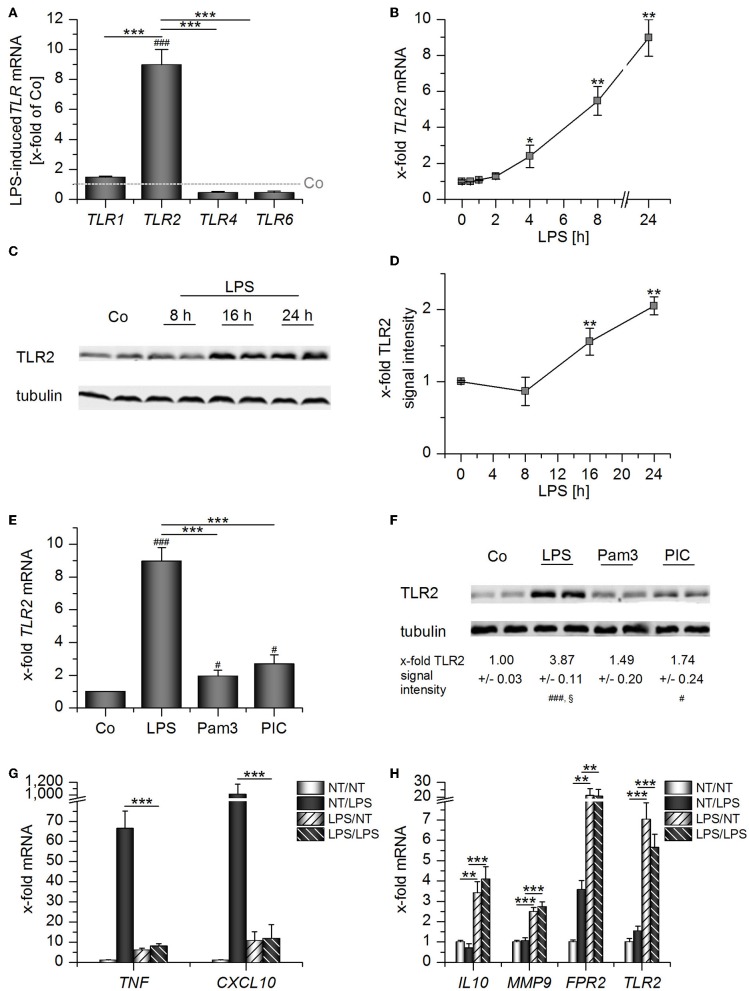
Long-Term LPS exposure upregulates TLR2 in AMs. **(A–D)** AMs were incubated with LPS (100 ng/mL) for 24 h **(A)** or the indicated time points **(B–D)**. TLR expression was measured by qPCR **(A,B)** or Western blot **(C,D)**. **(A)** TLR expression was normalized to the TLR expression values for the respective vehicle-treated control which was set as 1 (indicated by the dotted line). **(E–F)** AMs were treated with LPS (100 ng/mL), Pam_3_CSK_4_ (Pam3, 100 ng/mL) or Poly(I:C) (PIC, 1 μg/mL) for 24 h. TLR2 expression was analyzed by qPCR **(E)** and Western blot **(F)**. **(G,H)** Long-Term LPS exposure results in LPS tolerance. AMs were pretreated with LPS (100 ng/mL) for 24 h and restimulated with LPS (1 μg/mL) for 2 h. *TNF, CXCL10, IL10, MMP9, FPR2*, and *TLR2* mRNA expression levels were determined by qPCR. NT/NT, not treated; NT/LPS, LPS stimulation without pretreatment; LPS/NT, LPS pretreatment only; LPS/LPS, LPS pretreatment followed by LPS stimulation. Data from at least three independent experiments performed in duplicate with cells from different donors are shown and are presented as x-fold of solvent-treated cells ± SEM. ^#^*p* < 0.05, ^*###*^*p* < 0.001 vs. untreated cells, ^*^p < 0.05, ^**^p < 0.01, ^***^p < 0.001 as indicated, ^§^*p* < 0.05 vs. Pam3-treated cells. *p*-values were generated with ANOVA and Bonferroni's *post-hoc* test.

Prolonged exposure to LPS, which often occurs in sepsis, can result in LPS tolerance in macrophages and monocytes, thereby contributing to immunosuppression (20). Thus, we wondered whether TLR2 levels might be altered by LPS stimulation. LPS treatment for 24 h potently induced *TLR2*, but not *TLR1, TLR4*, and *TLR6* mRNA expression in AMs (Figure 3A). *TLR2* mRNA and TLR2 protein upregulation were most evident at later time points after LPS addition (Figures 3B–D). Other TLR ligands, i.e., the TLR2 ligand Pam_3_CSK_4_ and the TLR3 ligand Poly(I:C), also induced TLR2, although to a lesser extent (Figures 3E,F). LPS-mediated TLR2 upregulation was accompanied by LPS tolerance, as indicated by the inability of LPS-primed AMs to produce the inflammatory cytokines *TNF* and *CXCL10* in response to repeated LPS stimulation (Figure 3G). As seen in whole blood samples from sepsis patients (Figure 2), *TLR2* induction correlated with the overexpression of genes associated with immunosuppression (*IL10, FPR2*) or wound healing (*MMP9*) (Figure 3H).

**Figure 4:**
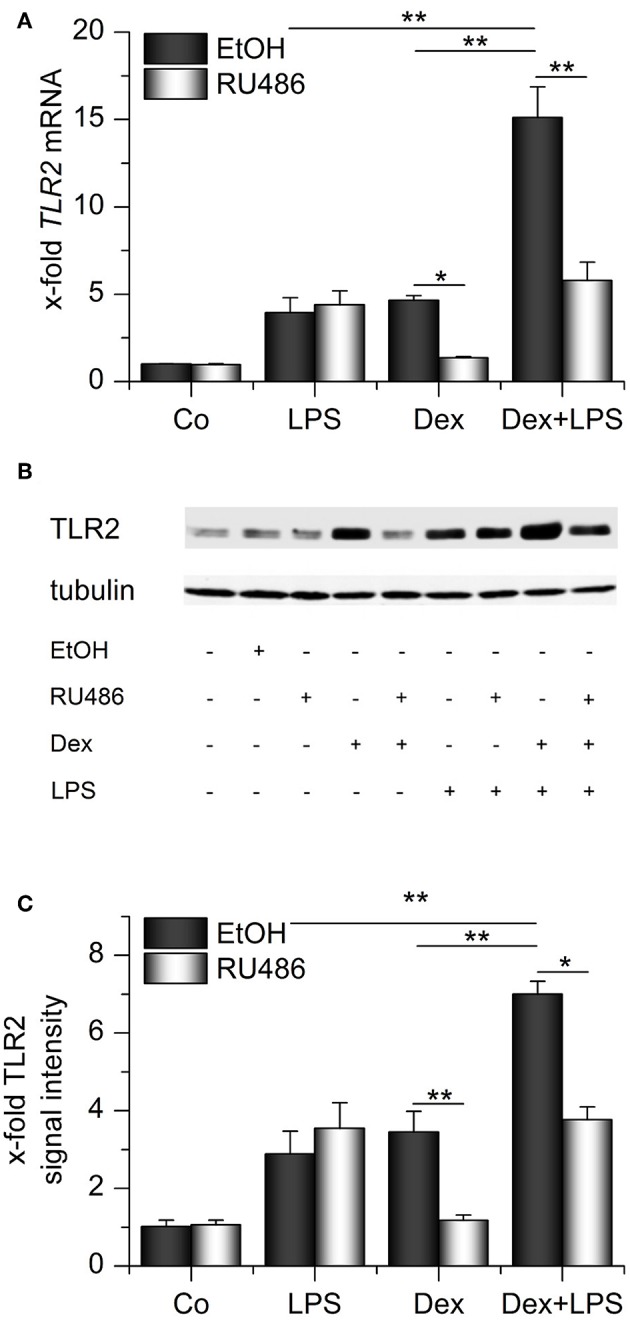
Synergistic upregulation of TLR2 by dexamethasone and LPS. AMs were preincubated with the GR inhibitor RU486 (10 μM) or solvent control (0.1% EtOH) and treated with LPS (100 ng/mL), Dex (1 μM) or both for 24 h. TLR2 expression was measured by qPCR (A) or Western blot **(B,C)**. **(B)** Representative blot. **(C)** Densitometric analysis. TLR2 signal intensities were quantified, normalized to tubulin values, and expressed as x-fold of untreated cells. **(A,C)** Data from at least three independent experiments performed in duplicate with cells from different donors are presented as means + SEM. ^*^p < 0.05, ^**^p < 0.01. *p*-values were generated by ANOVA with Bonferroni's *post-hoc* test.

We next evaluated whether the presence of both GCs and LPS might elevate TLR2 expression even further. Indeed, we observed that both compounds cooperatively induced TLR2 mRNA and protein (Figures 4A–C). Binding of Dex to its receptor was required for the cooperative regulation of TLR2 because the GR antagonist RU486 blocked Dex-induced effects both in the absence or presence of LPS. LPS-mediated upregulation of TLR2 was not affected by RU486 administration (Figures 4A–C).

**Figure 5:**
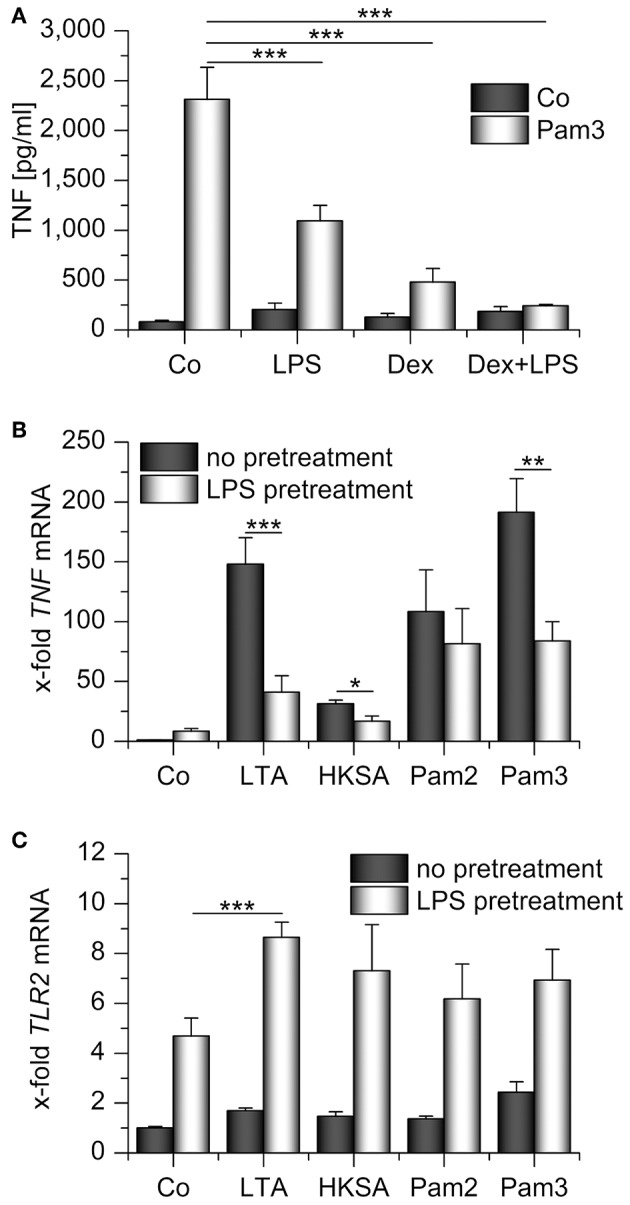
Impaired response toward TLR2 ligands in LPS- and/or Dex-pretreated AMs. **(A)** AMs were preincubated with LPS (100 ng/mL), Dex (1 μM), or both for 24 h and treated with Pam_3_CSK_4_ (Pam3, 1 μg/mL, 4 h). TNF secretion was assessed by TNF bioassay. **(B,C)** Primary human AMs were incubated with LPS (100 ng/mL, 24 h) before restimulation with TLR2 ligands for 2 h. LTA: lipoteichoic acid (5 μg/mL), HKSA: heat-killed *S. aureus* (10^8^ cells/mL), Pam2: Pam_2_CSK_4_ (1 μg/mL), Pam3: Pam_3_CSK_4_ (1 μg/mL). *TNF* **(B)** and *TLR2* **(C)** mRNA levels were determined by qPCR. Data from at least three independent experiments performed in duplicate with cells from different donors are presented as means + SEM. ^*^p < 0.05, ^**^p < 0.01, ^***^p < 0.001. *p*-values were generated by ANOVA with Bonferroni‘s *post-hoc* test.

We hypothesized that TLR2 upregulation might rescue TLR2 signaling in otherwise immunocompromised AMs. Therefore, we treated AMs pretreated with LPS, Dex, or a combination of both with the TLR2 ligand Pam_3_CSK_4_ and measured TNF levels in AM supernatants. None of the pretreatment schemes sensitized AMs toward Pam_3_CSK_4_. Quite in contrast, TLR2 signaling was inhibited in each of the conditions tested (Figure 5A). Similar effects were observed when we used different TLR2 ligands, i.e., lipoteichoic acid (LTA) and heat-killed *Staphylococcus aureus* (HKSA), to stimulate LPS-tolerant AMs. The response to the TLR1/6 ligand Pam_2_CSK_4_ showed a comparable tendency, but the reaction to this ligand was heterogenous amongst cells from different donors (Figure 5B). Interestingly, LTA treatment even enhanced TLR2 induction in LPS-pretreated AMs (Figure 5C). The lack of responsiveness toward TLR2 ligands in AMs that highly expressed TLR2 suggested an entirely different function of TLR2 in this context.

### TLR2 in Macrophage Supernatants

**Figure 6:**
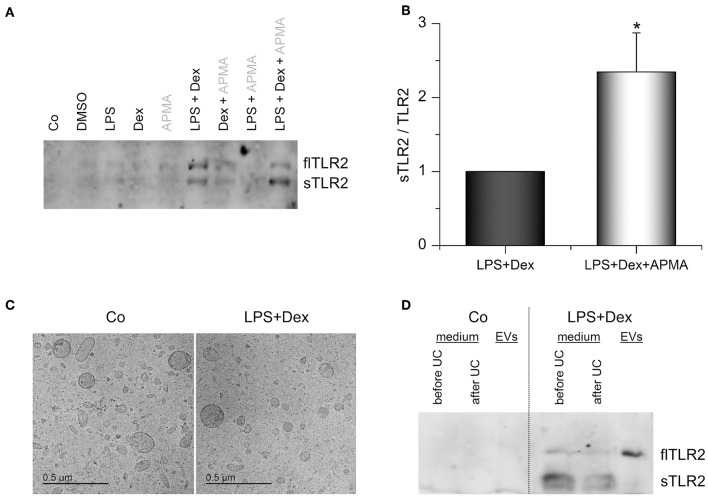
TLR2 in AM supernatants. **(A,B)** AMs were incubated with solvent control (0.1% DMSO), LPS (100 ng/mL), Dex (1 μM), or LPS+Dex for 24 h. 4-aminophenylmercuric acetate (APMA, 10 μM) was added to the indicated samples 5 h before supernatants were harvested. Soluble TLR2 (sTLR2) and full-length TLR2 (flTLR2) were detected in the supernatants by Western blot. **(A)** representative blot. **(B)** Relative sTLR2/TLR2 signal intensities are presented as means + SEM (*n* = 5). ^*^p < 0.05 (Student's *t*-test). **(C,D)** Cells were either left untreated (Co) or treated with LPS (100 ng/mL) + Dex (1 μM) for 3 days, and EVs were isolated by sequential centrifugation. **(C)** Representative cryo-TEM images of EVs from untreated (Co) and LPS+Dex-treated cells. **(D)** Representative Western blot analysis for TLR2 in AM supernatants before and after ultracentrifugation (UC) and in EVs is shown (*n* = 5).

We hypothesized that the upregulated membrane-bound TLR2 might serve as a precursor for sTLR2, known to antagonize TLR2-dependent cell actions. Supernatants of LPS+Dex-primed AMs indeed contained the soluble 83 kDa form of TLR2, as indicated by Western blot analysis (Figure 6A). As previously shown by Langjahr et al. (17), activation of metalloproteinases by 4-aminophenylmercuric acetate (APMA) resulted in enhanced sTLR2 shedding (Figure 6B). sTLR2 is produced via proteolytic cleavage of the TLR2 trans-membrane protein by ADAM10 and ADAM17 (17). These ADAMs were also expressed by alveolar macrophages, and *ADAM17* was even induced when LPS and Dex were present (Supplementary Figure 3). Thus, an involvement of ADAM17 in sTLR2 shedding is suggested.

Surprisingly, we also detected full-length TLR2 (flTLR2, ~ 102 kDa) and assumed that this might be due to the production of TLR2-containing extracellular vesicles (EVs). Therefore, EVs from macrophage supernatants were isolated by sequential centrifugation. Both untreated and LPS+Dex-treated cells produced vesicles of various sizes (50–300 nm) and mostly round in shape, as shown by cryo-TEM (Figure 6C). These vesicles were identified as the source of full-length TLR2 in macrophage supernatants, as indicated by Western blot analysis (Figure 6D).

### Vesicle Characterization

**Figure 7:**
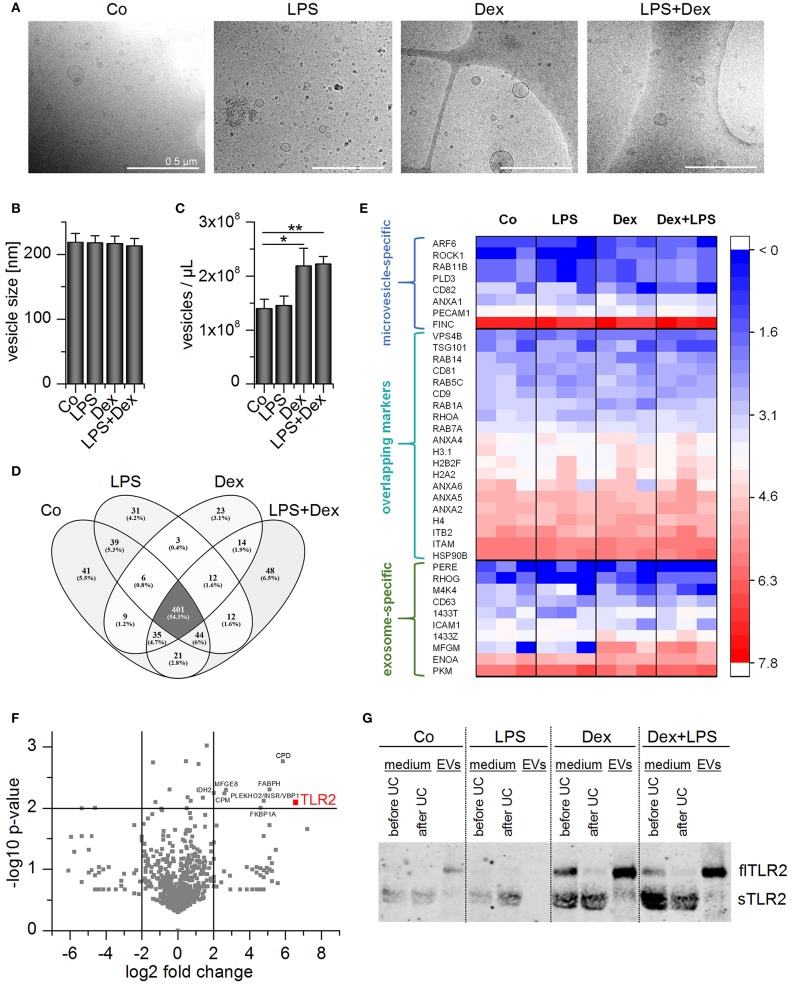
Characterization of THP-1 vesicles. Cells were incubated with medium only (Co), LPS (100 ng/mL), Dex (1 μM), or LPS+Dex for 72 h and EVs were isolated by sequential centrifugation. **(A)** Vesicles were visualized by cryo-TEM. Scale bar = 500 nm. **(B,C)** Average EV size **(B)** and concentration **(C)** were determined by nanoparticle tracking analysis. Data are presented as means + SEM (*n* = 7). ^*^p < 0.05, ^**^p < 0.01. *p*-values were generated by Mann–Whitney *U*-test. **(D,E)** EVs originating from differentially treated THP-1 cells were subjected to proteomics analysis (*n* = 3). **(D)** Overlap of identified proteins among treatment groups. **(E)** TLR2 and EV marker distribution. Log2 values of unique spectrum counts are shown for all three independent preparations per treatment. **(F)** Volcano plot of *p*-value vs. fold change in expression level in EV_LPS+Dex_ vs. EV_Co_. Proteins that were upregulated at least 4-fold in EV_LPS+Dex_ vs. EV_Co_ with a *p* < 0.01 are highlighted. **(G)** Representative Western blot result for TLR2 detection in THP-1 supernatant before and after ultracentrifugation (UC) and in EV fractions (*n* = 3).

For vesicle characterization and functional analysis, we used differentiated THP-1 cells as an easily accessible EV source. THP-1-derived EVs were similar to AM-derived EVs regarding size and shape (Figure 7A). Nanoparticle tracking analysis (NTA) was used to determine the EV size and concentration. Treatment schemes did not influence the vesicle size (~220 nm), but the number of vesicles slightly increased with Dex- or LPS+Dex-treatment (Figures 7B,C). This was not due to increased apoptosis, as determined by caspase-3 activity (Supplementary Figure 1). Vesicle numbers correlated with protein concentrations of the vesicle preparations (Supplementary Figure 4).

EV preparations were analyzed by high-resolution tandem mass spectrometry (MS/MS) to determine whether the treatment scheme had an impact on vesicle composition. A total of 709 proteins was detected in each of the independent experiments, and 401 proteins occurred in all four EV types (Figures 7D,E). The preparations did not show differences in vesicle marker abundance, and both exosome- and microvesicle-specific markers (36, 37) were detected (Figure 7E). Several proteins were enriched in EVs from LPS+Dex-treated cells (EV_LPS+Dex_), including TLR2 (Figure 7F). As seen in AM-derived EVs, TLR2 was most abundant in EV_LPS+Dex_ preparations when compared with other treatment schemes (Figure 7G).

**Figure 8:**
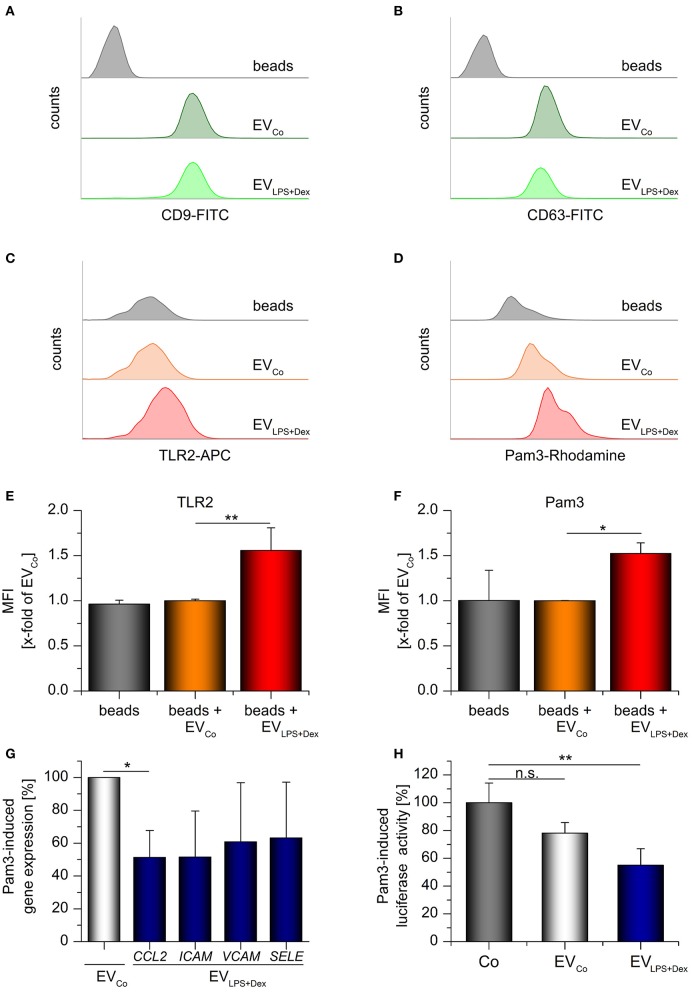
TLR2-containing vesicles act as decoy receptors. Differentiated THP-1 cells were incubated with medium only (Co) or LPS (100 ng/mL) + Dex (1 μM) for 72 h and EVs (EV_Co_ and EV_LPS+Dex_, respectively) were isolated by sequential centrifugation. **(A–F)** Bead-bound EVs from untreated or LPS+Dex-treated THP-1 cells were analyzed by flow cytometry. Unloaded latex beads served as controls. Histograms show bead counts vs. log fluorescence intensity. **(A,B)** EV loading was confirmed by staining for vesicle markers CD9 **(A)** and CD63 **(B)**. Representative histograms are shown (*n* = 3). **(C–F)** TLR2 staining and binding of rhodamine-labeled Pam_3_CSK_4_ (Pam3). **(C,D)** Representative histograms. **(E,F)** Mean fluorescence intensities were expressed as x-fold of EV_Co_ values + SEM (*n* = 3, duplicates). **(G)** Pam3-induced gene expression in HUVECs was measured by qRT-PCR. Pam3 was preincubated with the specified vesicles for 30 min at 37°C (2 × 10^10^ EVs/μg Pam3), and HUVECs were treated with the Pam3/EV mix (2 × 10^10^ EVs and 1 μg Pam3/mL) for 3 h. Data from 3 independent THP-1 vesicle preparations and HUVEC donors are presented as a percentage of EV_Co_-treated cells + SEM. H: Pam3-induced *CXCL8* promoter-dependent luciferase activity was quantified in hTLR2 HEK-Dual cells. Cells were either treated with Pam3 only (1 ng/mL, Co) or co-treated with Pam3 (1 ng/mL) and the specified vesicles (5 × 10^9^ EVs/ml) for 24 h. The Pam3/vesicle mix was preincubated for 30 min at 37°C before it was added to the cells. Data are expressed as percentage of Co-values + SEM (*n* = 4, duplicates). ^*^p < 0.05, ^**^p < 0.01, n.s.: not significant. *P*-values were generated by ANOVA with Bonferroni‘s *post-hoc* test or Student's *t*-test.

THP-1-derived EVs were further analyzed by flow cytometry. To this end, vesicles were coupled to aldehyde/sulfate latex beads. The presence of vesicle markers, tetraspanins CD9 and CD63 (36, 38), indicated that the vesicles were attached to the beads (Figures 8A,B). TLR2 staining confirmed that TLR2 was present in EV_LPS+Dex_ samples, but not in preparations from vehicle-treated cells (EV_Co_) (Figures 8C,E). In addition, staining of bead/EV complexes with fluorochrome-labeled Pam_3_CSK_4_ showed that EV_LPS+Dex_ were able to bind the TLR2 ligand, whereas EVs_Co_ were not (Figures 8D,F).

To examine the functional implications of TLR2-EV production, we treated primary human umbilical vein endothelial cells (HUVECs) with a mix of Pam_3_CSK_4_ and either EV_Co_ or EV_LPS+Dex_. Subsequently, the expression of the chemokine *CCL2* and the adhesion molecules *ICAM, VCAM*, and *SELE* were measured. Pam_3_CSK_4_-induced *CCL2* expression was decreased in HUVECs treated with ECV_LPS+Dex_ (Figure 8G). The expression of the three adhesion molecules tended to be reduced, although not significantly so due to high inter-individual differences between donors. Additionally, TLR2-responsive HEK reporter cells expressing a luciferase reporter gene under the control of the *CXCL8* promoter were used to study the influence of EVs on Pam_3_CSK_4_-induced inflammatory responses. We found indeed that TLR2-containing vesicles derived from LPS+Dex-treated cells were able to inhibit Pam_3_CSK_4_-induced luciferase production (Figure 8H). In summary, these data suggest that TLR2-EVs can exert decoy functions.

## Discussion

AMs are one of the first lines of defense against the invasion of airborne pathogens. The stimulation of TLRs triggers the production of proinflammatory cytokines, which in turn activate the hypothalamic-pituitary axis to induce the synthesis and secretion of anti-inflammatory GCs by the adrenal cortex, thereby limiting inflammation (39). Therapy of pulmonary diseases, such as asthma and chronic obstructive pulmonary disease, with inhaled GCs mimics the effects of endogenous GCs, resulting in decreased production of pro-inflammatory mediators by AMs (12).

Paradoxically, glucocorticoids have also been suggested to enhance inflammation and innate immune responses, particularly by upregulating TLR2 (40). An increase in TLR2 expression after GC administration was observed in many cell types, including epithelial cells (41–43), keratinocytes (44, 45), dendritic cells (10), and macrophages (11, 12, 45). Several studies showed that TLR ligands or inflammatory cytokines cooperate with GCs to induce TLR2 (12, 41–45). In line with our findings, Ji et al. (12) reported that coadministration of the GC budesonide and LPS resulted in elevated *TLR2* mRNA levels in human AMs, whereas *TLR4* was not affected.

Different mechanisms were suggested to underly the cooperative induction of TLR2 by GCs and pro-inflammatory stimuli. For example, *Haemophilus influenzae*-mediated TLR2 upregulation was enhanced by GCs *via* negative cross-talk with the mitogen-activated protein kinase (MAPK) p38 (41). Likewise, GC-mediated TLR2 induction was reported to depend on p38 inhibition *via* the GC-inducible phosphatase DUSP1 in keratinocytes and epithelial cells (44, 46). An entirely different mechanism was suggested to drive TLR2 expression in TNF-α/GC-treated A549 cells, requiring the collective recruitment of NF-κB, signal transducer and activator of transcription (STAT) transcription factors, and the GR to the *TLR2* promoter (42). In our hands, Dex-mediated TLR2 induction was GR-dependent and accompanied by DUSP1 induction (Supplementary Figure 5A). High DUSP1 expression levels correlated with the repression of p38 phosphorylation (Supplementary Figures 5B,C), suggesting that Dex-induced p38 inhibition may indeed play a role in TLR2 upregulation. However, direct binding of GC/GR complex to the *TLR2* promoter might also contribute to the overall effect.

Although GC-induced TLR2 upregulation has been suggested to enhance inflammation, a link between TLR2 induction and enhanced TLR2 responsiveness in immune cells has not been shown so far. In contrast, TLR2 upregulation has been reported to be paralleled by immunosuppression (10, 12), which was confirmed by our study. The lack of cytokine release upon TLR2 stimulation of GC-treated cells has been explained by the downstream blockade of the TLR2 receptor signaling and lack of TLR2 heterodimerization partners (10, 12, 40, 45).

In addition to GC treatment, we showed that long-term LPS exposure results in elevated TLR2 levels. Similar to GC-mediated effects, chronic exposure to LPS represses pro-inflammatory macrophage responses to recurring stimulation with LPS or other TLR ligands. TLR signaling is inhibited on many levels upon constant LPS stimulation, including downregulation of TLR adapter molecules and upregulation of anti-inflammatory factors (3, 20). In accordance, increased TLR2 expression did not lead to improved TLR2-mediated inflammatory responses in LPS-primed AMs in our study. Thus, we speculated that TLR2 induction by LPS and/or Dex might have anti-inflammatory effects, e.g., by sTLR2 release.

Secretion of sTLR2 balances responses to both viral and bacterial infections by binding a wide range of PAMPs and DAMPs, thereby inhibiting the activation of cellular TLR2 (16). We observed that sTLR2 was indeed produced by AMs, in particular after LPS+Dex-treatment. sTLR2 was enriched after activation of MPs, indicating that ectodomain shedding led to sTLR2 production (17).

In addition, we detected an unexpected protein that resembled full-length TLR2. In a previous study, Langjahr et al. (17) also observed a full-size TLR2 glycoprotein in human macrophage supernatant and hypothesized that it might correspond to the full-length protein associated with membrane vesicles. This hypothesis is supported by our results showing that flTLR2 is present in isolated extracellular vesicles (EVs). Of note, quantification of sTLR2 by ELISA, as used to analyze plasma samples from LPS-exposed volunteers or septic patients (18, 19), would also detect vesicular flTLR2. Thus, it presently remains elusive whether sTLR2, flTLR2, or both are present in the circulation in response to LPS or sepsis. Elevated sTLR2 plasma levels have been suggested as a biomarker for infections (18, 19). Therefore, it might be interesting to investigate whether TLR2-EVs might serve as diagnostic markers to assess disease progression, as currently discussed for various types of EVs (47).

Under physiological and pathological conditions, almost all cell types release cell-derived phospholipid-based bilayer membrane vesicles equipped with functional surface and membrane proteins and encapsulating various cargoes, including proteins, cytokines, lipids, and nucleic acids (48, 49). They are categorized as exosomes, microvesicles (MVs), and apoptotic bodies based on their size, pathway of formation, and membrane composition (49). Exosomes, which are 30–200 nm in size, derive from the late endosome. In contrast, MVs are between 100 and 1,000 nm in diameter and are formed through outward budding of the plasma membrane. Apoptotic bodies derived from apoptotic cells are very heterogeneous in size and morphology, and are, therefore, different from the other two EV subtypes (36, 50). Since exosomes and microvesicles display a similar appearance and composition as well as an overlapping size distribution, it is difficult to define their origin once isolated (36). Thus, we made no further distinction between these vesicle types in this work.

Flow cytometric analysis confirmed the presence of TLR2 in EV preparations derived from LPS+Dex-treated AMs and indicated an intact ligand binding ability. The overall inhibitory function of these vesicles suggests that they may act as a decoy, as previously shown for sTLR2 (17, 51). This decoy activity may involve competition for not only the microbial ligand but also the heterodimerization partners (51). Further studies are required to elucidate the anti-inflammatory potential of TLR2-containing EVs. These investigations might comprise more complex *in vitro* (52) or *in vivo* models (53, 54).

In summary, we showed for the first time that sTLR2 and full-length TLR2 are released by macrophages under anti-inflammatory conditions. Our data suggest that vesicle-bound flTLR2 has decoy functions, which may contribute to immunosuppression induced by GCs and chronic infections.

## Data Availability

The mass spectrometry proteomics datasets generated for this study have been been deposited to the ProteomeXchange Consortium via the PRIDE partner repository with the dataset identifier PXD013977 and 10.6019/PXD013977. In addition, the publicly available datasets analyzed in this study were obtained from Gene Expression Omnibus (GEO), accession numbers GSE4607 and GSE8121.

## Ethics Statement

Human lung tissue was obtained from patients undergoing lung resection. The use of human material was reviewed and approved by the local ethics committee (State Medical Board of Registration, Saarland, Germany; permission no. 213/06). The informed consent of all participating subjects was obtained.

## Author Contributions

JH, AD, RL, CD, AB, CF-T, MK, AK, and AKK designed, performed, and analyzed the experiments. JH wrote the paper. All authors contributed to drafting the manuscript. GF and HH provided materials and discussed the data. All authors read and approved the final manuscript.

### Conflict of Interest Statement

The authors declare that the research was conducted in the absence of any commercial or financial relationships that could be construed as a potential conflict of interest.

## Abbreviations

ACTB: beta actin
ADAM: a disintegrin and metalloproteinase
AFC: 7-amino-4-trifluoromethylcoumarin
AMs: alveolar macrophages
ANXA1: Annexin A1
AP-1: activator protein-1
APC: allophycocyanine
CCL: CC-chemokine ligand
COX2: cyclooxygenase-2
CXCL: C–X–C motif ligand
Dex: dexamethasone
DMEM: Dulbecco's modified Eagle medium
DUSP1: dual-specificity phosphatase-1
EVs: extracellular vesicles
FCS: fetal calf serum
FITC: fluorescein isothiocyanate
flTLR: full length TLR
FPR2: formyl peptide receptor 2
GC: glucocorticoid
GILZ: glucocorticoid-induced leucine zipper
HEK: human embryonic kidney
HKSA: heat-killed *Staphylococcus aureus*
ICAM1: intercellular adhesion molecule 1
IL: interleukin
IL1RN: IL1 receptor antagonist
IFN: interferon
IRFs: interferon regulatory factors
LPS: lipopolysaccharide
LTA: lipoteichoic acid
MMP: matrix metalloproteinase
NF-κB: nuclear factor-κB
NOS2: nitric oxide synthase 2
NTA: nanoparticle tracking analysis
MAPK: mitogen-activated protein kinase
PE: phycoerythrin
MARCO: macrophage receptor with collagenous structure
PMA: phorbol 12-myristate 13-acetate
Poly(I:C): polyinosinic:polycytidylic acid
PRR: pattern recognition receptor
RPMI: Roswell Park Memorial Institute
SELE: selectin E
STAT: signal transducer and activator of transcription
sTLR: soluble TLR
TCA: trichloroacetic acid
TEM: transmission electron microscopy
TNF: tumor necrosis factor
TLR: toll-like receptor
VCAM1: vascular cell adhesion molecule 1
VCAN: versican.

## References

[1] Vandevyver S Dejager L Tuckermann J Libert C. New insights into the anti-inflammatory mechanisms of glucocorticoids: an emerging role for glucocorticoid-receptor-mediated transactivation. Endocrinology. (2013) 154:993–1007. 10.1210/en.2012-2045 23384835

[2] Ayroldi E Macchiarulo A Riccardi C. Targeting glucocorticoid side effects: selective glucocorticoid receptor modulator or glucocorticoid-induced leucine zipper? A perspective. FASEB J. (2014) 28:5055–70. 10.1096/fj.14-254755 25205742

[3] Hoppstädter J Kiemer AK Glucocorticoid-induced leucine zipper (GILZ) in immuno suppression: master regulator or bystander? Oncotarget. (2015) 6:38446–57. 10.18632/oncotarget.6197 26498359 PMC4770713

[4] Hoppstädter J Diesel B Zarbock R Breinig T Monz D Koch M. Differential cell reaction upon Toll-like receptor 4 and 9 activation in human alveolar and lung interstitial macrophages. Respir Res. (2010) 11:124. 10.1186/1465-9921-11-124 20843333 PMC2949727

[5] Hussell T Bell TJ. Alveolar macrophages: plasticity in a tissue-specific context. Nat Rev Immunol. (2014) 14:81–93. 10.1038/nri3600 24445666

[6] Medzhitov R. Toll-like receptors and innate immunity. Nat Rev Immunol. (2001) 1:135–45. 10.1038/35100529 11905821

[7] Netea MG van der Meer JW. Immunodeficiency and genetic defects of pattern-recognition receptors. N Engl J Med. (2011) 364:60–70. 10.1056/NEJMra1001976 21208109

[8] Cao X. Self-regulation and cross-regulation of pattern-recognition receptor signalling in health and disease. Nat Rev Immunol. (2016) 16:35–50. 10.1038/nri.2015.8 26711677

[9] Kawasaki T Kawai T. Toll-like receptor signaling pathways. Front Immunol. (2014) 5:461. 10.3389/fimmu.2014.00461 25309543 PMC4174766

[10] Rozkova D Horvath R Bartunkova J Spisek R. Glucocorticoids severely impair differentiation and antigen presenting function of dendritic cells despite upregulation of Toll-like receptors. Clin Immunol. (2006) 120:260–71. 10.1016/j.clim.2006.04.567 16765091

[11] Olivares-Morales MJ De La Fuente MK Dubois-Camacho K Parada D Diaz-Jimenez D Torres-Riquelme A. Glucocorticoids impair phagocytosis and inflammatory response against crohn's disease-associated adherent-invasive *Escherichia coli*. Front Immunol. (2018) 9:1026. 10.3389/fimmu.2018.01026 29867993 PMC5964128

[12] Ji J von Scheele I Billing B Dahlen B Lantz AS Larsson K. Effects of budesonide on toll-like receptor expression in alveolar macrophages from smokers with and without COPD. Int J Chron Obstruct Pulmon Dis. (2016) 11:1035–43. 10.2147/COPD.S102668 27274225 PMC4876676

[13] van Bergenhenegouwen J Plantinga TS Joosten LA Netea MG Folkerts G Kraneveld AD. TLR2 and Co: a critical analysis of the complex interactions between TLR2 and coreceptors. J Leukoc Biol. (2013) 94:885–902. 10.1189/jlb.0113003 23990624

[14] LeBouder E Rey-Nores JE Rushmere NK Grigorov M Lawn SD Affolter M. Soluble forms of Toll-like receptor (TLR)2 capable of modulating TLR2 signaling are present in human plasma and breast milk. J Immunol. (2003) 171:6680–9. 10.4049/jimmunol.171.12.6680 14662871

[15] Liew FY Xu D Brint EK O'Neill LA. Negative regulation of toll-like receptor-mediated immune responses. Nat Rev Immunol. (2005) 5:446–58. 10.1038/nri1630 15928677

[16] Henrick BM Yao XD Taha AY German JB Rosenthal KL. Insights into soluble toll-like receptor 2 as a downregulator of virally induced inflammation. Front Immunol. (2016) 7:291. 10.3389/fimmu.2016.00291 27531999 PMC4969314

[17] Langjahr P Diaz-Jimenez D De la Fuente M Rubio E Golenbock D Bronfman FC. Metalloproteinase-dependent TLR2 ectodomain shedding is involved in soluble toll-like receptor 2 (sTLR2) production. PLoS ONE. (2014) 9:e104624. 10.1371/journal.pone.0104624 25531754 PMC4273945

[18] Ten Oever J Kox M van de Veerdonk FL Mothapo KM Slavcovici A Jansen TL. The discriminative capacity of soluble Toll-like receptor (sTLR)2 and sTLR4 in inflammatory diseases. BMC Immunol. (2014) 15:55. 10.1186/s12865-014-0055-y 25406630 PMC4240815

[19] Holst B Szakmany T Raby AC Hamlyn V Durno K Hall JE. Soluble Toll-like receptor 2 is a biomarker for sepsis in critically ill patients with multi-organ failure within 12 h of ICU admission. Intensive Care Med Exp. (2017) 5:2. 10.1186/s40635-016-0116-z 28092080 PMC5236041

[20] Biswas SK Lopez-Collazo E. Endotoxin tolerance: new mechanisms, molecules and clinical significance. Trends Immunol. (2009) 30:475–87. 10.1016/j.it.2009.07.009 19781994

[21] Li CC Munitic I Mittelstadt PR Castro E Ashwell JD. Suppression of dendritic cell-derived IL-12 by endogenous glucocorticoids is protective in LPS-induced sepsis. PLoS Biol. (2015) 13:e1002269. 10.1371/journal.pbio.1002269 26440998 PMC4595142

[22] Hoppstädter J Diesel B Eifler LK Schmid T Brüne B Kiemer AK. Glucocorticoid-induced leucine zipper is downregulated in human alveolar macrophages upon Toll-like receptor activation. Eur J Immunol. (2012) 42:1282–93. 10.1002/eji.201142081 22539300

[23] Hoppstädter J Diesel B Linnenberger R Hachenthal N Flamini S Minet M. Amplified host defense by toll-like receptor-mediated downregulation of the glucocorticoid-induced leucine zipper (GILZ) in macrophages. Front Immunol. (2019) 9:3111. 10.3389/fimmu.2018.03111 30723476 PMC6349698

[24] Hoppstädter J Seif M Dembek A Cavelius C Huwer H Kraegeloh A. M2 polarization enhances silica nanoparticle uptake by macrophages. Front Pharmacol. (2015) 6:55. 10.3389/fphar.2015.00055 25852557 PMC4369656

[25] Hahn RT Hoppstädter J Hirschfelder K Hachenthal N Diesel B Kessler SM. Downregulation of the glucocorticoid-induced leucine zipper (GILZ) promotes vascular inflammation. Atherosclerosis. (2014) 234:391–400. 10.1016/j.atherosclerosis.2014.03.028 24747114

[26] Ziaei A Hoppstädter J Kiemer AK Ramezani M Amirghofran Z Diesel B. Inhibitory effects of teuclatriol, a sesquiterpene from salvia mirzayanii, on nuclear factor-kappaB activation and expression of inflammatory mediators. J Ethnopharmacol. (2015) 160:94–100. 10.1016/j.jep.2014.10.041 25446581

[27] Weber NC Blumenthal SB Hartung T Vollmar AM Kiemer AK. ANP inhibits TNF-alpha-induced endothelial MCP-1 expression–involvement of p38 MAPK and MKP-1. J Leukoc Biol. (2003) 74:932–41. 10.1189/jlb.0603254 12960255

[28] Hoppstädter J Kessler SM Bruscoli S Huwer H Riccardi C Kiemer AK. Glucocorticoid-induced leucine zipper: a critical factor in macrophage endotoxin tolerance. J Immunol. (2015) 194:6057–67. 10.4049/jimmunol.1403207 25964494

[29] Hoppstädter J Hachenthal N Valbuena-Perez JV Lampe S Astanina K Kunze MM. Induction of Glucocorticoid-induced Leucine Zipper (GILZ) contributes to anti-inflammatory effects of the natural product curcumin in macrophages. J Biol Chem. (2016) 291:22949–60. 10.1074/jbc.M116.733253 27629417 PMC5087716

[30] Théry C Amigorena S Raposo G Clayton A. Isolation and characterization of exosomes from cell culture supernatants and biological fluids. Curr Protoc Cell Biol. (2006) 3:22. 10.1002/0471143030.cb0322s30 18228490

[31] Fecher-Trost C Wissenbach U Beck A Schalkowsky P Stoerger C Doerr J. The *in vivo* TRPV6 protein starts at a non-AUG triplet, decoded as methionine, upstream of canonical initiation at AUG. J Biol Chem. (2013) 288:16629–44. 10.1074/jbc.M113.469726 23612980 PMC3675598

[32] Perez-Riverol Y Csordas A Bai J Bernal-Llinares M Hewapathirana S Kundu DJ. The PRIDE database and related tools and resources in 2019: improving support for quantification data. Nucleic Acids Res. (2019) 47:D442–50. 10.1093/nar/gky1106 30395289 PMC6323896

[33] Dembek A Laggai S Kessler SM Czepukojc B Simon Y Kiemer AK. Hepatic interleukin-6 production is maintained during endotoxin tolerance and facilitates lipid accumulation. Immunobiology. (2017) 222:786–96. 10.1016/j.imbio.2017.01.003 28132721

[34] Wong HR Shanley TP Sakthivel B Cvijanovich N Lin R Allen GL. Genome-level expression profiles in pediatric septic shock indicate a role for altered zinc homeostasis in poor outcome. Physiol Genomics. (2007) 30:146–55. 10.1152/physiolgenomics.00024.2007 17374846 PMC2770262

[35] Cvijanovich N Shanley TP Lin R Allen GL Thomas NJ Checchia P. Validating the genomic signature of pediatric septic shock. Physiol Genomics. (2008) 34:127–34. 10.1152/physiolgenomics.00025.2008 18460642 PMC2440641

[36] van Niel G D'Angelo G Raposo G. Shedding light on the cell biology of extracellular vesicles. Nat Rev Mol Cell Biol. (2018) 19:213–28. 10.1038/nrm.2017.125 29339798

[37] Jeppesen DK Fenix AM Franklin JL Higginbotham JN Zhang Q Zimmerman LJ. Reassessment of exosome composition. Cell. (2019) 177:428–445.e418. 10.1016/j.cell.2019.02.029 30951670 PMC6664447

[38] Thery C Witwer KW Aikawa E Alcaraz MJ Anderson JD Andriantsitohaina R. Minimal information for studies of extracellular vesicles 2018 (MISEV2018): a position statement of the International Society for Extracellular Vesicles and update of the MISEV2014 guidelines. J Extracell Vesicles. (2018) 7:1535750. 10.1080/20013078.2018.1535750 30637094 PMC6322352

[39] Chinenov Y Rogatsky I. Glucocorticoids and the innate immune system: crosstalk with the toll-like receptor signaling network. Mol Cell Endocrinol. (2007) 275:30–42. 10.1016/j.mce.2007.04.014 17576036

[40] Cruz-Topete D Cidlowski JA. One hormone, two actions: anti- and pro-inflammatory effects of glucocorticoids. Neuroimmunomodulation. (2015) 22:20–32. 10.1159/000362724 25227506 PMC4243162

[41] Shuto T Imasato A Jono H Sakai A Xu H Watanabe T. Glucocorticoids synergistically enhance nontypeable Haemophilus influenzae-induced Toll-like receptor 2 expression via a negative cross-talk with p38 MAP kinase. J Biol Chem. (2002) 277:17263–70. 10.1074/jbc.M112190200 11867630

[42] Hermoso MA Matsuguchi T Smoak K Cidlowski JA. Glucocorticoids and tumor necrosis factor alpha cooperatively regulate toll-like receptor 2 gene expression. Mol Cell Biol. (2004) 24:4743–56. 10.1128/MCB.24.11.4743-4756.2004 15143169 PMC416411

[43] Homma T Kato A Hashimoto N Batchelor J Yoshikawa M Imai S. Corticosteroid and cytokines synergistically enhance toll-like receptor 2 expression in respiratory epithelial cells. Am J Respir Cell Mol Biol. (2004) 31:463–9. 10.1165/rcmb.2004-0161OC 15242847

[44] Shibata M Katsuyama M Onodera T Ehama R Hosoi J Tagami H Glucocorticoids enhance Toll-like receptor 2 expression in human keratinocytes stimulated with Propionibacterium acnes or proinflammatory cytokines. J Invest Dermatol. (2009) 129:375–82. 10.1038/jid.2008.237 18704103

[45] Su Q Pfalzgraff A Weindl G. Cell type-specific regulatory effects of glucocorticoids on cutaneous TLR2 expression and signalling. J Steroid Biochem Mol Biol. (2017) 171:201–8. 10.1016/j.jsbmb.2017.03.023 28377308

[46] Sakai A Han J Cato AC Akira S Li JD. Glucocorticoids synergize with IL-1beta to induce TLR2 expression via MAP Kinase Phosphatase-1-dependent dual Inhibition of MAPK JNK and p38 in epithelial cells. BMC Mol Biol. (2004) 5:2. 10.1186/1471-2199-5-2 15125785 PMC419700

[47] Roy S Hochberg FH Jones PS. Extracellular vesicles: the growth as diagnostics and therapeutics; a survey. J Extracell Vesicles. (2018) 7:1438720. 10.1080/20013078.2018.1438720 29511461 PMC5827771

[48] Fuhrmann G Herrmann IK Stevens MM. Cell-derived vesicles for drug therapy and diagnostics: opportunities and challenges. Nano Today. (2015) 10:397–409. 10.1016/j.nantod.2015.04.004 28458718 PMC5409525

[49] Yanez-Mo M Siljander PR Andreu Z Zavec AB Borras FE Buzas EI. Biological properties of extracellular vesicles and their physiological functions. J Extracell Vesicles. (2015) 4:27066. 10.3402/jev.v4.27066 25979354 PMC4433489

[50] Goes A Fuhrmann G. Biogenic and biomimetic carriers as versatile transporters to treat infections. ACS Infect Dis. (2018) 4:881–92. 10.1021/acsinfecdis.8b00030 29553240

[51] Raby AC Le Bouder E Colmont C Davies J Richards P Coles B. Soluble TLR2 reduces inflammation without compromising bacterial clearance by disrupting TLR2 triggering. J Immunol. (2009) 183:506–17. 10.4049/jimmunol.0802909 19542461

[52] Susewind J de Souza Carvalho-Wodarz C Repnik U Collnot E-M Schneider-Daum N Griffiths GW. A 3D co-culture of three human cell lines to model the inflamed intestinal mucosa for safety testing of nanomaterials. Nanotoxicology. (2016) 10:53–62. 10.3109/17435390.2015.1008065 25738417

[53] Kordelas L Rebmann V Ludwig AK Radtke S Ruesing J Doeppner TR. MSC-derived exosomes: a novel tool to treat therapy-refractory graft-versus-host disease. Leukemia. (2014) 28:970–3. 10.1038/leu.2014.41 24445866

[54] Lai P Weng J Guo L Chen X Du X. Novel insights into MSC-EVs therapy for immune diseases. Biomarker Res. (2019) 7:6. 10.1186/s40364-019-0156-0 30923617 PMC6423844

