# Supplementary material for: Toll-Like Receptor 2 Release by Macrophages: An Anti-inflammatory Program Induced by Glucocorticoids and Lipopolysaccharide
Source: Front Immunol. 2019 Jul 23;10:1634. doi: 10.3389/fimmu.2019.01634 (PMC6664002; doi:10.3389/fimmu.2019.01634)
Supplement: Supplementary file 1 [file Data_Sheet_1.pdf]

Supplementary Material

Toll-like receptor 2 release by macrophages: an anti-inflammatory program induced by glucocorticoids and lipopolysaccharide

Jessica Hoppstädter[1*], Anna Dembek[1], Rebecca Linnenberger[1], Charlotte Dahlem[1], Ahmad Barghash[2], Claudia Fecher-Trost[3], Gregor Fuhrmann[4], Marcus Koch[5], Annette Kraegeloh[5], Hanno Huwer[6], Alexandra K. Kiemer[1*]

[1]Department of Pharmacy, Pharmaceutical Biology, Saarland University, Saarbrücken, Germany

[2]Department of Computer Science, German Jordanian University, Amman, Jordan

[3]Department of Experimental and Clinical Pharmacology and Toxicology, Saarland University, Homburg/Saar, Germany

[4]Helmholtz Institute for Pharmaceutical Research Saarland, Saarbrücken, Germany

[5]INM – Leibniz Institute for New Materials, Saarbrücken, Germany

[6]Department of Cardiothoracic Surgery, Völklingen Heart Centre, Völklingen, Germany

*Corresponding authors:

j.hoppstaedter@mx.uni-saarland.de, pharm.bio.kiemer@mx.uni-saarland.de

Supplementary Figures

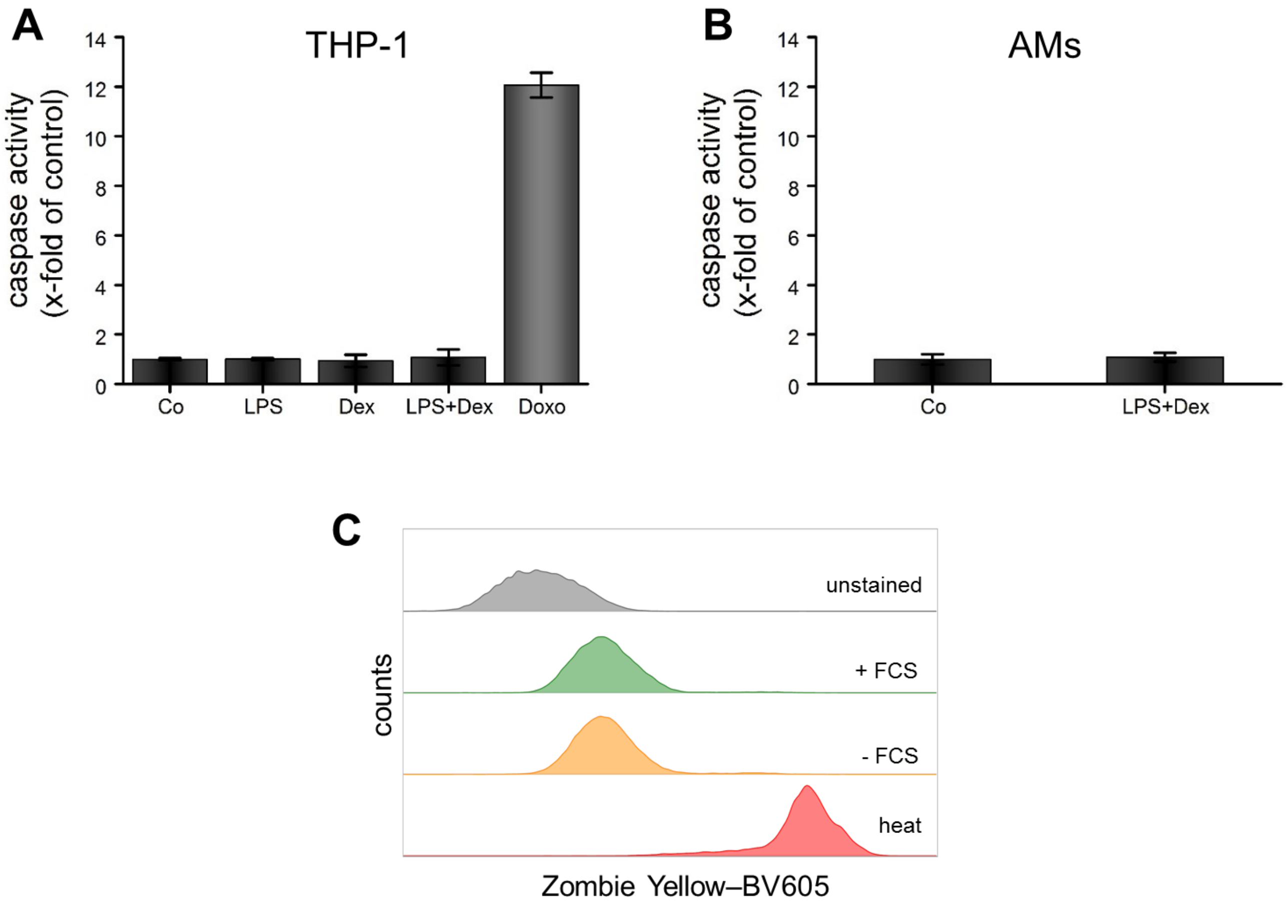

Supplementary Figure 1. *Treatment schemes and serum deprivation do not impair cell viability.* A, B: Caspase-3-like activity assay. Cells were treated with the vehicle control (0.1% DMSO, Co), LPS (100 ng/mL), Dex (1 µM), or LPS + Dex for 3 d in serum-free medium. Treatment with 10 µM doxorubicin (Doxo) for 24 h served as a positive control. An increase in apoptosis-asscociated caspase-3-like activity was neither detected in THP-1 cells (A) nor in AMs (B) (n = 3). C: Zombie yellow viability staining. Differentiated THP-1 cells were cultured in in serum-containing (+ FCS) or serum-free (- FCS) medium for 3 d. Cells incubated at 60°C for 1 h (heat) served as a positive control. Cell viability was not affected by serum-free medium (n = 2, triplicates).

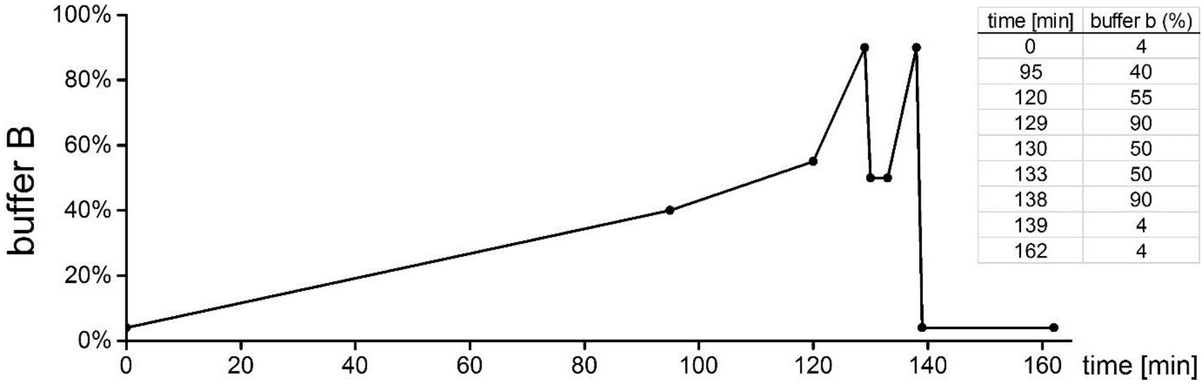

time [min]	buffer b (%)
0	4
95	40
120	55
129	90
130	50
133	50
138	90
139	4
162	4

Supplementary Figure 2. *Gradient for nano-liquid chromatography.* Time points for changes in buffer B concentrations are given in the table.

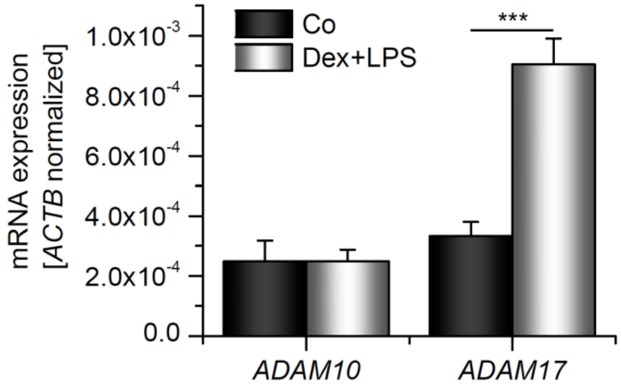

Supplementary Figure 3: ADAM10 *and* ADAM17 *expression in AMs*. AMs were treated with vehicle (Co) or LPS (100 ng/ml) + Dex (1 µM) for 24 h. *ADAM10* and *ADAM17* expression was determined by qPCR, and values were normalized to the housekeeping gene *ACTB*. The means of 3 experiments performed in triplicate + SEM are shown. ***$p < 0.001$. p-values were generated by ANOVA with Bonferroni's post-hoc test.

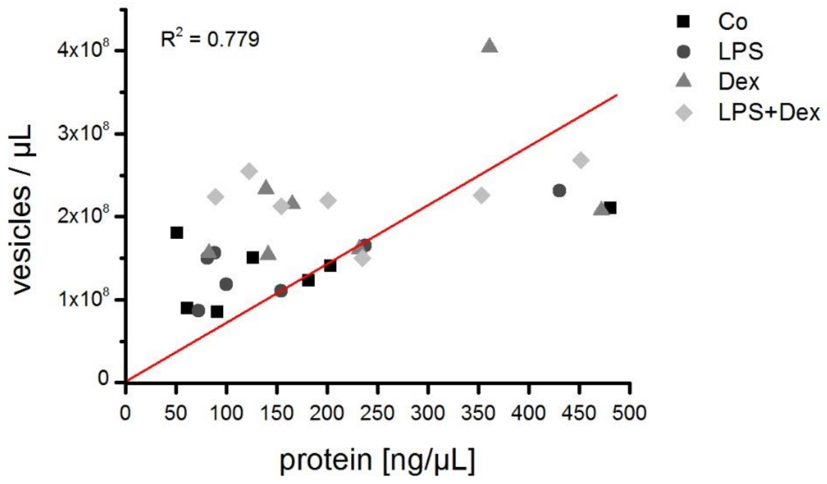

Supplementary Figure 4: *Correlation between EV concentration and protein amount.* EVs were isolated from THP-1 macrophages treated with the vehicle control (0.1% DMSO, Co), LPS (100 ng/mL), Dex (1 µM) or LPS + Dex for 3 d in serum-free medium. Values for seven individual isolations are given. ■ = Co, ● = LPS, ▲ = Dex and ♦ = LPS+Dex.

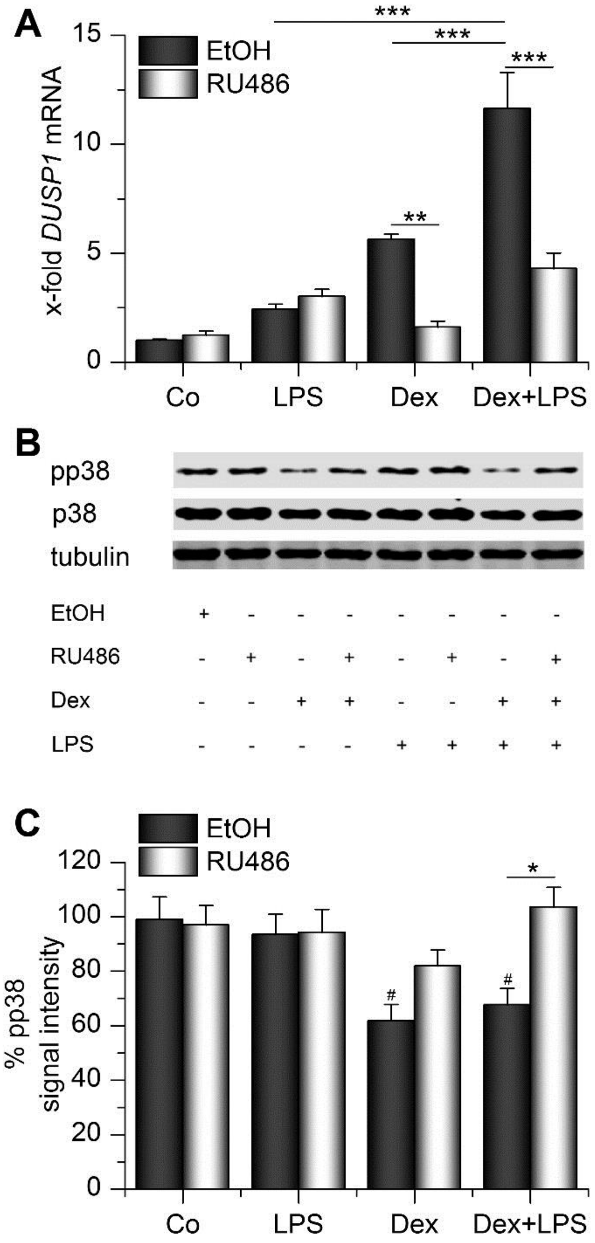

Supplementary Figure 5: *Influence of dexamethasone and LPS on* DUSP1 *expression and p38 MAPK activation.* AMs were preincubated with the GR inhibitor RU486 (10 µM) or solvent control (0.1% EtOH) and treated with LPS (100 ng/mL), Dex (1 µM), or both for 24 h. A: *DUSP1* expression was measured by qPCR. B, C: Phospho-p38 and total p38 were analyzed by Western Blot. Tubulin served as a loading control. Data from at least three independent experiments performed in duplicate with cells from different donors are presented as means + SEM and A, C: *p < 0.05, **p < 0.01. ***p < 0.001. C: #p < 0.05 vs. vehicle-treated Co. p-values were generated by ANOVA with Bonferroni's post-hoc test.

Supplementary Tables

Supplementary Table 1: PCR conditions.

Gene	Sequence (5'→3') forward primer	Sequence (5'→3') reverse primer	Probe sequence (5'FAM→3'BHQ)	Probe [nM]	$MgCl_2$ [mM]	Annealing [°C]
ACTB	TGCGTGACATTAAGGAGAAG	GTCAGGCAGCTCGTAGCTCT	CACGGCTGCTTCCAGCTCCTC	60	4	60
ADAM10	TGCCCAGATATCCAGTCATGTT	TCACCATGAAACTGATGTTACGG	no probe	N/A	N/A	60
ADAM17	AGAGAACCACCTGAAGAGCTTG	TCCCCTCTGCCCATGTATCT	no probe	N/A	N/A	60
CCL2	TTGATGTTTTAAGTTTATCTTTCATGG	CAGGGGTAGAACTGTGGTTCA	no probe	N/A	N/A	60
CXCL10	GAGCCTACAGCAGAGGAACC	AAGGCAGCAAATCAGAATCG	TCCAGTCTCAGCACCATGAATCAAA	60	4	60
DUSP1	CAGCTGCTGCAGTTTGAGTC	AGGTAGCTCAGCGCACTGTT	no probe	N/A	N/A	64
FPR2	GCATCCTCAGGAAAATGCACC	GCATCCTCAGGAAAATGCACC	no probe	N/A	N/A	60
ICAM	GAAGTGGCCCTCCATAGACA	TCAAGGGTTGGGGTCAGTAG	no probe	N/A	N/A	60
IL10	CAACAGAAGCTTCCATTCCA	AGCAGTTAGGAAGCCCCAAG	AGCCTGACCACGCTTTCTAGCTGTTGAG	100	4	60

MMP9	CTTTGAGTC CGGTGGACG AT	TCGCCAGTA CTTCCCATC CT	no probe	N/A	N/A	60
SELE	AGCCCAGAG CCTTCAGTG TA	CCCTGCATG TCACAGCTT TA	no probe	N/A	N/A	60
TNF	CTCCACCCA TGTGCTCCT CA	CTCTGGCAG GGGCTCTTG AT	CACCATCAGCCG CATCGCCGTCTC	100	3	60
TLR1	AGCAAAGAA ATAGATTAC ACATCA	TTACCTACA TCATACACT CAAAT	ATTCCTCCTGTT GATATTGCTTTTG	60	5	57
TLR2	GCAAGCTGC GGAAGATAA TG	CGCAGCTCT CAGATTTAC CC	ATGGACGAGGCT CAGCGGGAAG	60	3	60
TLR4	ATGAAATGA GTTGCAGCA GA	AGCCATCTG TGTCTCCCT AA	AAGTGATGTTTG ATGGACCTCTGA ATCT	60	4	58
TLR6	TTTACTTGG ATGATGGTG AATAGT	AGTTCCCCA GATGAAACA TT	GTCGTAAGTAAC TGTCTGGAGGTGC	100	5	57
VCAM	CGAGACCAC CCCAGAATC TA	CTGTGGTGC TGCAAGTCA AT	no probe	N/A	N/A	60

Supplementary Table 2: Antibodies and TLR2 ligands for flow cytometry.

antibody / ligand	amount per sample	order no.	supplier
**FITC anti-human CD9, Mouse IgG1, kappa [HI9a 25]**	0.5 µg	BLD-312103	Biozol
**FITC anti-human CD63, Mouse IgG1, [H5C6]**	1 µg	BLD-353005	Biozol
**FITC Mouse IgG1, kappa Isotype Ctrl (FC) [MOPC-21]**	1 µg	BLD-400109	Biozol
**APC anti-human TLR2 (CD282) Mouse IgG2a [TL2.1]**	2 µg	17-9922-41	ThermoFisher
**APC Mouse IgG2a kappa Isotype Control [eBM2a]**	2 µg	17-4724-81	ThermoFisher
**Rhodamine-conjugated Pam_3CSK_4**	0.5 µg	tlrl-rpms	Invivogen
